# A Constrained Talagrand Transportation Inequality with Applications to Rate-Distortion-Perception Theory

**DOI:** 10.3390/e27040441

**Published:** 2025-04-19

**Authors:** Li Xie, Liangyan Li, Jun Chen, Lei Yu, Zhongshan Zhang

**Affiliations:** 1 School of Information and Electronics, Beijing Institute of Technology, Beijing 100081, China; lixie.lx@gmail.com; 2 Department of Electrical and Computer Engineering, McMaster University, Hamilton, ON L8S 4K1, Canada; lil61@mcmaster.ca; 3 School of Statistics and Data Science, LPMC, KLMDASR, and LEBPS, Nankai University, Tianjin 300071, China; leiyu@nankai.edu.cn; 4 School of Cyberspace Science and Technology, Beijing Institute of Technology, Beijing 100081, China; zhangzs@bit.edu.cn

**Keywords:** Kullback–Leibler divergence, optimal transport, rate-distortion-perception theory, squared error, transportation inequality, Wasserstein distance

## Abstract

A constrained version of Talagrand’s transportation inequality is established, which reveals an intrinsic connection between the Gaussian distortion-rate-perception functions with limited common randomness under the Kullback–Leibler divergence-based and squared Wasserstein-2 distance-based perception measures. This connection provides an organizational framework for assessing existing bounds on these functions. In particular, we show that the best-known bounds of Xie et al. are nonredundant when examined through this connection.

## 1. Introduction

Traditional rate-distortion theory [1] seeks to determine the minimum rate required to encode a source while ensuring that the expected distortion remains below a given threshold. However, minimizing distortion alone does not always align with human perception, particularly in applications like image and audio compression, where perceptual quality plays a crucial role. Rate-distortion-perception theory addresses this by introducing a perception constraint [2], measured by a divergence between the source and reconstruction distributions, to ensure that the reconstructed signal remains perceptually similar to the original. This framework enables a more nuanced tradeoff between compression efficiency, signal fidelity, and perceptual quality, making it particularly relevant in modern machine learning and generative model-based compression techniques. The origin of rate-distortion-perception theory can be traced back to the foundational work of Klejsa et al. [3,4] and Saldi et al. [5,6] on distribution-preserving quantization. However, it was arguably the influential paper by Blau and Michaeli [7] (see also [8,9]) that brought the theory to the forefront of the research community’s attention. Since then, the field has developed rapidly, offering insights into architectural design principles [10,11,12,13], the role of randomness [14,15,16,17], and fundamental performance limits [18,19,20,21,22]. These advances have also catalyzed a variety of new research directions and applications [23,24,25,26,27,28,29,30,31,32].

Kullback–Leibler divergence and squared Wasserstein-2 distance are among the most widely adopted perception measures. When the source distribution is Gaussian, these two measures are intrinsically linked through Talagrand’s transportation inequality [33]. Exploring the implications of this connection in rate-distortion-perception theory is of significant interest. The availability of partial knowledge of the reconstruction distribution in perception-aware lossy source coding, in turn, motivates the study of constrained versions of Talagrand’s transportation inequality. Such inequalities will further strengthen the link between the information-theoretic performance limits under these two perception measures.

The main contributions of this paper are as follows:

1. We prove a variant of Talagrand’s transportation inequality, where the reference distribution is Gaussian and the other distribution is subject to constraints on its first- and second-order statistics.
2. This inequality is then used to establish a connection between the Gaussian distortion-rate-perception functions with limited common randomness under the Kullback–Leibler divergence-based and squared Wasserstein-2 distance-based perception measures. We leverage this connection as an organizational framework to assess existing bounds on these functions. In particular, it is shown that the best-known bounds of Xie et al. [22] are nonredundant when examined through this connection.

The rest of this paper is organized as follows. Section 2 presents a constrained Talagrand’s transportation inequality. Its application to rate-distortion-perception theory is explored in Section 3. We conclude this paper in Section 4.

We adopt standard notations for information measures, e.g., h(·) for differential entropy and I(·;·) for mutual information. For a given random variable *X*, its distribution, mean, and variance are written as $p_{X}$, $\mu_{X}$, and $\sigma_{X}^{2}$, respectively. A Gaussian distribution with mean μ and variance $\sigma^{2}$ is denoted by $N(\mu ,\sigma^{2})$. We use $\Pi (p_{X},p_{\hat{X}})$ to represent the set of all possible joint distributions with marginals $p_{X}$ and $p_{\hat{X}}$. The cardinality of set S is expressed as |S|. For a real number *a*, define ${\left(a\right)}_{+}:=\max\left\{a,0\right\}$. Throughout this paper, the logarithm function is assumed to have a base *e*.

## 2. A Constrained Talagrand Transportation Inequality

For $p_{X}=N\left(\mu_{X},\sigma_{X}^{2}\right)$, Talagrand’s transportation inequality [33] states that

(1) $$W_{2}^{2}\left(p_{X},p_{\hat{X}}\right)\leq 2\sigma_{X}^{2}\phi_{KL}\left(p_{\hat{X}}∥p_{X}\right),$$

where

(2) $$\phi \left(p_{\hat{X}}∥p_{X}\right):=E\left[\log\frac{p_{\hat{X}}\left(\hat{X}\right)}{p_{X}\left(\hat{X}\right)}\right]$$

is the Kullback–Leibler divergence and

(3) $$W_{2}^{2}\left(p_{X},p_{\hat{X}}\right):=\underset{p_{X\hat{X}}\in \Pi \left(p_{X},p_{\hat{X}}\right)}{\inf}E\left[{\left(X-\hat{X}\right)}^{2}\right]$$

is the squared Wasserstein-2 distance. Note that Talagrand’s transportation inequality does not impose any assumptions on $p_{\hat{X}}$. However, in practice, we often have partial knowledge of $p_{\hat{X}}$, which can be exploited to strengthen the inequality. In this paper, we focus on the case where $p_{\hat{X}}$ satisfies $\mu_{\hat{X}}=\mu_{X}$ and $\sigma_{\hat{X}}\leq \sigma_{X}$. Under these constraints on $p_{\hat{X}}$, we establish the following variant of Talagrand’s transportation inequality:

**Theorem** **1.** *For $p_{X}=N\left(\mu_{X},\sigma_{X}^{2}\right)$ and $p_{\hat{X}}$ with $\mu_{\hat{X}}=\mu_{X}$ and $\sigma_{\hat{X}}\leq \sigma_{X}$,*

(4) $$W_{2}^{2}\left(p_{X},p_{\hat{X}}\right)\leq 2\sigma_{X}^{2}\left(1-e^{-\phi_{KL}\left(p_{\hat{X}}∥p_{X}\right)}\right).$$

It is clear that (4) is stronger than (1) since $1+z\leq e^{z}$ for z∈R. To prove Theorem 1, we need the following well-known result (see, e.g., Propositions 1 and 2 [22]) concerning the Gaussian extremal property of the Kullback–Leibler divergence and the squared Wasserstein-2 distance.

**Lemma** **1.** *For $p_{X}=N\left(\mu_{X},\sigma_{X}^{2}\right)$ and $p_{\hat{X}}$ with $E[{\hat{X}}^{2}]<\infty$,*

(5) $$\begin{array}{cc} \phi_{KL}\left(p_{\hat{X}}∥p_{X}\right) & \geq \phi_{KL}\left(p_{{\hat{X}}^{G}}∥p_{X}\right)\\ & =\log\frac{\sigma_{X}}{\sigma_{\hat{X}}}+\frac{{\left(\mu_{X}-\mu_{\hat{X}}\right)}^{2}+\sigma_{\hat{X}}^{2}-\sigma_{X}^{2}}{2\sigma_{X}^{2}} \end{array}$$

*and*

(6) $$\begin{array}{cc} W_{2}^{2}\left(p_{X},p_{\hat{X}}\right) & \geq W_{2}^{2}\left(p_{X},p_{{\hat{X}}^{G}}\right)\\ \; & ={\left(\mu_{X}-\mu_{\hat{X}}\right)}^{2}+{\left(\sigma_{X}-\sigma_{\hat{X}}\right)}^{2}, \end{array}$$

*where $p_{{\hat{X}}^{G}}:=N\left(\mu_{\hat{X}},\sigma_{\hat{X}}^{2}\right)$.*

Lemma 1 indicates that when the reference distribution is Gaussian, replacing the other distribution with its Gaussian counterpart leads to reductions in both the Kullback–Leibler divergence and the squared Wasserstein-2 distance. These reductions turn out to be quantitatively related, as shown by the next result.

**Lemma** **2.** *For $p_{X}=N\left(\mu_{X},\sigma_{X}^{2}\right)$ and $p_{\hat{X}}$ with $E[{\hat{X}}^{2}]<\infty$,*

(7) $$W_{2}^{2}\left(p_{X},p_{\hat{X}}\right)-W_{2}^{2}\left(p_{X},p_{{\hat{X}}^{G}}\right)\leq 2\sigma_{X}\sigma_{\hat{X}}\left(1-e^{-(\phi_{KL}\left(p_{\hat{X}}∥p_{X}\right)-\phi_{KL}\left(p_{{\hat{X}}^{G}}∥p_{X}\right))}\right).$$

**Proof of Lemma** **2.** Note that

(8) $$\begin{array}{cc} W_{2}^{2}\left(p_{X},p_{\hat{X}}\right) & ={\left(\mu_{X}-\mu_{\hat{X}}\right)}^{2}+W_{2}^{2}\left(p_{X-\mu_{X}},p_{\hat{X}-\mu_{\hat{X}}}\right)\\ \; & ={\left(\mu_{X}-\mu_{\hat{X}}\right)}^{2}+\sigma_{X}^{2}W_{2}^{2}\left(p_{\sigma_{X}^{-1}\left(X-\mu_{X}\right)},p_{\sigma_{X}^{-1}\left(\hat{X}-\mu_{\hat{X}}\right)}\right)\\ \; & \overset{\left(a\right)}{\leq }{\left(\mu_{X}-\mu_{\hat{X}}\right)}^{2}+\sigma_{X}^{2}+\sigma_{\hat{X}}^{2}-2\sigma_{X}^{2}\sqrt{\frac{1}{2\pi e}e^{2h(\sigma_{X}^{-1}\hat{X})}}\\ \; & \overset{\left(b\right)}{=}W_{2}^{2}\left(p_{X},p_{{\hat{X}}^{G}}\right)+2\sigma_{X}\sigma_{\hat{X}}-2\sigma_{X}^{2}\sqrt{\frac{1}{2\pi e}e^{2h(\sigma_{X}^{-1}\hat{X})}}, \end{array}$$

where (*a*) is due to (Equation (8), [34]) and (*b*) is due to Lemma 1. Moreover,

(9) $$\begin{array}{cc} h(\sigma_{X}^{-1}\hat{X}) & =h\left(\hat{X}\right)-\log\sigma_{X}\\ \; & =\frac{1}{2}\log\frac{2\pi e\sigma_{\hat{X}}^{2}}{\sigma_{X}^{2}}-\phi_{KL}\left(p_{\hat{X}}∥p_{X}\right)+\phi_{KL}\left(p_{{\hat{X}}^{G}}∥p_{X}\right). \end{array}$$

Substituting (9) into (8) proves Lemma 2. □

**Proof of Theorem** **1.** In view of Lemmas 1 and 2,

(10) $$\begin{array}{cc} W_{2}^{2}\left(p_{X},p_{\hat{X}}\right) & \leq \max_{\mu ,\sigma }\eta \left(\mu ,\sigma \right) \end{array}$$

(11) $$\begin{array}{cc} \mathrm{subject}\mathrm{to} & \;\mu =\mu_{X}, \end{array}$$

(12) $$\begin{array}{c} \sigma \leq \sigma_{X}, \end{array}$$

(13) $$\begin{array}{c} \frac{{\left(\mu_{X}-\mu \right)}^{2}}{2\sigma_{X}^{2}}+\psi \left(\sigma \right)\leq \phi_{KL}\left(p_{\hat{X}}∥p_{X}\right), \end{array}$$

where

(14) $$\eta \left(\mu ,\sigma \right):=-2\sigma_{X}^{2}e^{\frac{{\left(\mu_{X}-\mu \right)}^{2}+\sigma^{2}-\sigma_{X}^{2}}{2\sigma_{X}^{2}}-\phi_{KL}\left(p_{\hat{X}}∥p_{X}\right)}+{\left(\mu_{X}-\mu \right)}^{2}+\sigma_{X}^{2}+\sigma^{2}$$

and

(15) $$\psi \left(\sigma \right):=\log\frac{\sigma_{X}}{\sigma }+\frac{\sigma^{2}-\sigma_{X}^{2}}{2\sigma_{X}^{2}}.$$

Since ψ(σ) decreases monotonically from *∞* to 0 as σ varies from 0 to $\sigma_{X}$ and increases monotonically from 0 to *∞* as σ varies from $\sigma_{X}$ to *∞*, there must exist $\underline{\sigma }\leq \sigma_{X}$ and $\bar{\sigma }\geq \sigma_{X}$ satisfying

(16) $$\psi \left(\underline{\sigma }\right)=\psi \left(\bar{\sigma }\right)=\phi_{KL}\left(p_{\hat{X}}∥p_{X}\right).$$

Note that (10)–(13) can be written compactly as

(17) $$W_{2}^{2}\left(p_{X},p_{\hat{X}}\right)\leq \max_{\sigma \in [\underline{\sigma },\sigma_{X}]}\eta \left(\mu_{X},\sigma \right).$$

For $\sigma \in [\underline{\sigma },\sigma_{X}]$, we have

(18) $$\frac{\sigma^{2}-\sigma_{X}^{2}}{2\sigma_{X}^{2}}-\phi_{KL}\left(p_{\hat{X}}∥p_{X}\right)\leq 0,$$

and, consequently,

(19) $$\begin{array}{cc} \frac{\partial }{\partial \sigma }\eta \left(\mu_{X},\sigma \right) & =-2\sigma e^{\frac{\sigma^{2}-\sigma_{X}^{2}}{2\sigma_{X}^{2}}-\phi_{KL}\left(p_{\hat{X}}∥p_{X}\right)}+2\sigma \\ \; & \geq 0, \end{array}$$

which implies that the maximum in (17) is attained at $\sigma =\sigma_{X}$. Thus,

(20) $$\begin{array}{cc} W_{2}^{2}\left(p_{X},p_{\hat{X}}\right) & \leq \eta (\mu_{X},\sigma_{X})\\ \; & =2\sigma_{X}^{2}\left(1-e^{-\phi_{KL}\left(p_{\hat{X}}∥p_{X}\right)}\right). \end{array}$$

This proves Theorem 1. □

The following result shows that Talagrand’s transportation inequality (1) corresponds to a relaxed version of (10), obtained by removing Constraints (11) and (12).

**Theorem** **2.** *For $p_{X}=N\left(\mu_{X},\sigma_{X}^{2}\right)$ and $p_{\hat{X}}$ with $E[{\hat{X}}^{2}]<\infty$,*

(21) $$\begin{array}{cc} 2\sigma_{X}^{2}\phi_{KL}\left(p_{\hat{X}}∥p_{X}\right) & =\max_{\mu ,\sigma }\eta \left(\mu ,\sigma \right) \end{array}$$

(22) $$\begin{array}{cc} subject\;to & \;\frac{{\left(\mu_{X}-\mu \right)}^{2}}{2\sigma_{X}^{2}}+\psi \left(\sigma \right)\leq \phi_{KL}\left(p_{\hat{X}}∥p_{X}\right). \end{array}$$

**Proof of Theorem** **2.** First, recall the definitions of $\underline{\sigma }$ and $\bar{\sigma }$ from (16). It can be verified that

(23) $$\begin{array}{cc} \; & \frac{\partial }{\partial {\left(\mu_{X}-\mu \right)}^{2}}\eta \left(\mu ,\sigma \right)=-e^{\frac{{\left(\mu_{X}-\mu \right)}^{2}+\sigma^{2}-\sigma_{X}^{2}}{2\sigma_{X}^{2}}-\phi_{KL}\left(p_{\hat{X}}∥p_{X}\right)}+1. \end{array}$$

Given $\sigma <\underline{\sigma }$, there is no μ satisfying (22). Given $\sigma \in [\underline{\sigma },\sigma_{X}]$, for μ satisfying (22), we have

(24) $$\begin{array}{cc} \frac{{\left(\mu_{X}-\mu \right)}^{2}+\sigma^{2}-\sigma_{X}^{2}}{2\sigma_{X}^{2}}-\phi_{KL}\left(p_{\hat{X}}∥p_{X}\right) & =\frac{{\left(\mu_{X}-\mu \right)}^{2}}{2\sigma_{X}^{2}}+\psi \left(\sigma \right)-\phi_{KL}\left(p_{\hat{X}}∥p_{X}\right)-\log\frac{\sigma_{X}}{\sigma }\\ \; & \leq 0, \end{array}$$

and, consequently,

(25) $$\begin{array}{c} \frac{\partial }{\partial {\left(\mu_{X}-\mu \right)}^{2}}\eta \left(\mu ,\sigma \right)\geq 0, \end{array}$$

which implies that the maximum value of η(μ,σ) over μ satisfying (22) is attained when

(26) $$\begin{array}{c} \log\frac{\sigma_{X}}{\sigma }+\frac{{\left(\mu_{X}-\mu \right)}^{2}+\sigma^{2}-\sigma_{X}^{2}}{2\sigma_{X}^{2}}=\phi_{KL}\left(p_{\hat{X}}∥p_{X}\right). \end{array}$$

Therefore, for $\sigma \in [\underline{\sigma },\sigma_{X}]$,

(27) $$\begin{array}{c} \max_{\mu :(22)}\eta \left(\mu ,\sigma \right)=\kappa \left(\sigma \right), \end{array}$$

where

(28) $$\begin{array}{c} \kappa \left(\sigma \right):=2\sigma_{X}^{2}\left(\phi_{KL}\left(p_{\hat{X}}∥p_{X}\right)-\log\frac{\sigma_{X}}{\sigma }+1\right)-2\sigma_{X}\sigma . \end{array}$$

Since the maximum value of κ(σ) over $\sigma \in [\underline{\sigma },\sigma_{X}]$ is attained at $\sigma =\sigma_{X}$, it follows that

(29) $$\begin{array}{c} \max_{\sigma \in [\underline{\sigma },\sigma_{X}]}\max_{\mu :(22)}\eta \left(\mu ,\sigma \right)=2\sigma_{X}^{2}\phi_{KL}\left(p_{\hat{X}}∥p_{X}\right). \end{array}$$

Given $\sigma \in (\sigma_{X},\sqrt{2\sigma_{X}^{2}\phi_{KL}\left(p_{\hat{X}}∥p_{X}\right)+\sigma_{X}^{2}})$, for μ satisfying (22), we have

(30) $$\begin{array}{cc} \; & \frac{\partial }{\partial {\left(\mu_{X}-\mu \right)}^{2}}\eta \left(\mu ,\sigma \right)\left\{\begin{array}{cc} \geq 0 & \mathrm{if}\frac{{\left(\mu_{X}-\mu \right)}^{2}+\sigma^{2}-\sigma_{X}^{2}}{2\sigma_{X}^{2}}\leq \phi_{KL}\left(p_{\hat{X}}∥p_{X}\right),\\ <0 & \mathrm{if}\frac{{\left(\mu_{X}-\mu \right)}^{2}+\sigma^{2}-\sigma_{X}^{2}}{2\sigma_{X}^{2}}>\phi_{KL}\left(p_{\hat{X}}∥p_{X}\right), \end{array}\right. \end{array}$$

which implies that the maximum value of η(μ,σ) over μ satisfying (22) is attained when

(31) $$\frac{{\left(\mu_{X}-\mu \right)}^{2}+\sigma^{2}-\sigma_{X}^{2}}{2\sigma_{X}^{2}}=\phi_{KL}\left(p_{\hat{X}}∥p_{X}\right).$$

Therefore, for $\sigma \in (\sigma_{X},\sqrt{2\sigma_{X}^{2}\phi_{KL}\left(p_{\hat{X}}∥p_{X}\right)+\sigma_{X}^{2}})$,

(32) $$\max_{\mu :(22)}\eta \left(\mu ,\sigma \right)=2\sigma_{X}^{2}\phi_{KL}\left(p_{\hat{X}}∥p_{X}\right).$$

As a consequence,

(33) $$\max_{\sigma \in (\sigma_{X},\sqrt{2\sigma_{X}^{2}\phi_{KL}\left(p_{\hat{X}}∥p_{X}\right)+\sigma_{X}^{2}})}\max_{\mu :(22)}\eta \left(\mu ,\sigma \right)=2\sigma_{X}^{2}\phi_{KL}\left(p_{\hat{X}}∥p_{X}\right).$$

Given $\sigma \in [\sqrt{2\sigma_{X}^{2}\phi_{KL}\left(p_{\hat{X}}∥p_{X}\right)+\sigma_{X}^{2}},\bar{\sigma }]$, for μ satisfying (22), we have

(34) $$\frac{\partial }{\partial {\left(\mu_{X}-\mu \right)}^{2}}\eta \left(\mu ,\sigma \right)\leq 0,$$

which implies that the maximum value of η(μ,σ) over μ satisfying (22) is attained when

(35) $${\left(\mu_{X}-\mu \right)}^{2}=0,i.e.,\mu =\mu_{X}.$$

Therefore, for $\sigma \in [\sqrt{2\sigma_{X}^{2}\phi_{KL}\left(p_{\hat{X}}∥p_{X}\right)+\sigma_{X}^{2}},\bar{\sigma }]$,

(36) $$\max_{\mu :(22)}\eta \left(\mu ,\sigma \right)=\kappa^{\prime }\left(\sigma \right),$$

where

(37) $$\kappa^{\prime }\left(\sigma \right):=-2\sigma_{X}^{2}e^{\frac{\sigma^{2}-\sigma_{X}^{2}}{2\sigma_{X}^{2}}-\phi_{KL}\left(p_{\hat{X}}∥p_{X}\right)}+\sigma_{X}^{2}+\sigma^{2}.$$

Since the maximum value of $\kappa^{\prime }\left(\sigma \right)$ over $\sigma \in [\sqrt{2\sigma_{X}^{2}\phi_{KL}\left(p_{\hat{X}}∥p_{X}\right)+\sigma_{X}^{2}},\bar{\sigma }]$ is attained at $\sigma =\sqrt{2\sigma_{X}^{2}\phi_{KL}\left(p_{\hat{X}}∥p_{X}\right)+\sigma_{X}^{2}}$, it follows that

(38) $$\max_{\sigma \in [\sqrt{2\sigma_{X}^{2}\phi_{KL}\left(p_{\hat{X}}∥p_{X}\right)+\sigma_{X}^{2}},\bar{\sigma }]}\max_{\mu :(22)}\eta \left(\mu ,\sigma \right)=2\sigma_{X}^{2}\phi_{KL}\left(p_{\hat{X}}∥p_{X}\right).$$

Given $\sigma >\bar{\sigma }$, there is no μ satisfying (22). Combining (29), (33), and (38) proves Theorem 2. □

## 3. Application to Rate-Distortion-Perception Theory

A length-*n* perception-aware lossy source coding system consists of an encoder $f^{\left(n\right)}:R^{n}\times K\to J$, a decoder $g^{\left(n\right)}:J\times K\to R^{n}$, and a random seed *K*. It takes an i.i.d. source sequence $X^{n}$ as input and produces an i.i.d. reconstruction sequence ${\hat{X}}^{n}$. Specifically, the encoder maps $X^{n}$ and *K* to a codeword *J* in codebook J according to some conditional distribution $p_{J|X^{n}K}$, while the decoder generates ${\hat{X}}^{n}$ based on *J* and *K* according to some conditional distribution $p_{{\hat{X}}^{n}|JK}$. Here, *K* is assumed to be uniformly distributed over the alphabet K and independent of $X^{n}$. End-to-end distortion is quantified by $\frac{1}{n}\sum_{t=1}^{n}E\left[{\left(X_{t}-{\hat{X}}_{t}\right)}^{2}\right]$ and perceptual quality by $\frac{1}{n}\sum_{t=1}^{n}\phi \left(p_{X_{t}},p_{{\hat{X}}_{t}}\right)$ with some divergence ϕ. It is clear that $\frac{1}{n}\sum_{t=1}^{n}\phi \left(p_{X_{t}},p_{{\hat{X}}_{t}}\right)=\phi \left(p_{X},p_{\hat{X}}\right)$, where $p_{X}$ and $p_{\hat{X}}$ are the marginal distributions of $X^{n}$ and ${\hat{X}}^{n}$, respectively.

**Definition** **1.** *For an i.i.d. source ${\left\{X_{t}\right\}}_{t=1}^{\infty }$, distortion level D is said to be achievable and subject to the compression rate constraint R, the common randomness rate constraint $R_{c}$, and the perception constraint P if there exists a length-n perception-aware lossy source coding system such that*

(39) $$\begin{array}{cc} \; & \frac{1}{n}\log\left|J\right|\leq R, \end{array}$$

(40) $$\begin{array}{cc} \; & \frac{1}{n}\log\left|K\right|\leq R_{c}, \end{array}$$

(41) $$\begin{array}{cc} \; & \frac{1}{n}\sum_{t=1}^{n}E\left[{\left(X_{t}-{\hat{X}}_{t}\right)}^{2}\right]\leq D, \end{array}$$

(42) $$\begin{array}{cc} \; & \frac{1}{n}\sum_{t=1}^{n}\phi \left(p_{X_{t}},p_{{\hat{X}}_{t}}\right)\leq P. \end{array}$$

*Moreover, the reconstruction sequence ${\hat{X}}^{n}$ is ensured to be i.i.d. The infimum of such achievable distortion levels D is denoted by $D(R,R_{c},P|\phi )$.*

The following result, which is built upon (Theorem 1, [6]) (see also (Theorem 2, [15])), provides a single-letter characterization of $D(R,R_{c},P|\phi )$.

**Theorem** **3** (Theorem 1, [22])**.** *For $p_{X}$ with $E[X^{2}]<\infty$,*

(43) $$\begin{array}{cc} D(R,R_{c},P|\phi ) & =\underset{p_{U\hat{X}|X}}{\inf}E\left[{\left(X-\hat{X}\right)}^{2}\right] \end{array}$$

(44) $$\begin{array}{cc} subject\;to & \;X↔U↔\hat{X}\;form\;a\;Markov\;chain, \end{array}$$

(45) $$\begin{array}{cc} \; & \;I(X;U)\leq R, \end{array}$$

(46) $$\begin{array}{cc} \; & \;I\left(\hat{X};U\right)\leq R+R_{c}, \end{array}$$

(47) $$\begin{array}{cc} \; & \;\phi (p_{X},p_{\hat{X}})\leq P. \end{array}$$

According to (Lemmas 1 and 3, [22]), for $p_{X}=N\left(\mu_{X},\sigma_{X}^{2}\right)$, there is no loss of generality in focusing on $p_{\hat{X}}$ with $\mu_{\hat{X}}=\mu_{X}$ and $\sigma_{\hat{X}}\leq \sigma_{X}$ as far as $D(R,R_{c},P|\phi_{KL})$ and $D(R,R_{c},P|W_{2}^{2})$ are concerned; therefore, it follows from Theorem 1 that

(48) $$D\left(R,R_{c},2\sigma_{X}^{2}\left(1-e^{-P}\right)|W_{2}^{2}\right)\leq D\left(R,R_{c},P|\phi_{KL}\right).$$

This reveals an intrinsic connection between the Gaussian distortion-rate-perception functions with limited common randomness under the Kullback–Leibler divergence-based and squared Wasserstein-2 distance-based perception measures. Since $D(R,R_{c},P|\phi_{KL})$ and $D(R,R_{c},P|W_{2}^{2})$ do not appear to have explicit expressions, recent research efforts have been devoted to deriving bounds for these functions. Note that via (48), every lower bound on $D(R,R_{c},\cdot |W_{2}^{2})$ induces a corresponding lower bound on $D(R,R_{c},\cdot |\phi_{KL})$, and every upper bound on $D(R,R_{c},\cdot |\phi_{KL})$ induces a corresponding upper bound on $D(R,R_{c},\cdot |W_{2}^{2})$. As a consequence, a lower bound on $D(R,R_{c},\cdot |\phi_{KL})$ (or an upper bound on $D(R,R_{c},\cdot |W_{2}^{2})$) can be considered redundant if it is implied by a lower bound on $D(R,R_{c},\cdot |W_{2}^{2})$ (or an upper bound on $D(R,R_{c},\cdot |\phi_{KL})$) through this connection. This provides an organizational framework for assessing existing bounds on these functions. As an illustrative example, we examine the best-known bounds due to Xie et al. [22], summarized in the following two theorems, from this perspective.

Let

(49) $$\xi \left(R,R_{c}\right):=\sqrt{\left(1-e^{-2R}\right)\left(1-e^{-2(R+R_{c})}\right)}.$$

Moreover, let σ(P) be the unique number $\sigma \in [0,\sigma_{X}]$ satisfying ψ(σ)=P, where ψ(σ) is defined in (15).

**Theorem** **4** (Theorem 3, [22])**.** *For $p_{X}=N\left(\mu_{X},\sigma_{X}^{2}\right)$,*

(50) $$\underline{D}\left(R,R_{c},P|\phi_{KL}\right)\leq D\left(R,R_{c},P|\phi_{KL}\right)\leq \bar{D}\left(R,R_{c},P|\phi_{KL}\right),$$

*where*

(51) $$\begin{array}{cc} \; & \underline{D}\left(R,R_{c},P|\phi_{KL}\right)\\ \; & :=\min_{\sigma_{\hat{X}}\in \left[\sigma \left(P\right),\sigma_{X}\right]}\sigma_{X}^{2}+\sigma_{\hat{X}}^{2}-2\sigma_{X}\sigma_{\hat{X}}\sqrt{\left(1-e^{-2R}\right)\left(1-e^{-2(R+R_{c}+P-\psi \left(\sigma_{\hat{X}}\right))}\right)} \end{array}$$

*and*

(52) $$\bar{D}\left(R,R_{c},P|\phi_{KL}\right):=\sigma_{X}^{2}-\sigma_{X}^{2}\xi^{2}\left(R,R_{c}\right)+{\left(\sigma \left(P\right)-\sigma_{X}\xi \left(R,R_{c}\right)\right)}_{+}^{2}.$$

**Theorem** **5** (Theorem 4, [22])**.** *For $p_{X}=N\left(\mu_{X},\sigma_{X}^{2}\right)$,*

(53) $$\underline{D}\left(R,R_{c},P|W_{2}^{2}\right)\leq D\left(R,R_{c},P|W_{2}^{2}\right)\leq \bar{D}\left(R,R_{c},P|W_{2}^{2}\right),$$

*where*

(54) $$\begin{array}{cc} \; & \underline{D}\left(R,R_{c},P|W_{2}^{2}\right)\\ \; & :=\min_{\sigma_{\hat{X}}\in \left[{\left(\sigma_{X}-\sqrt{P}\right)}_{+},\sigma_{X}\right]}\sigma_{X}^{2}+\sigma_{\hat{X}}^{2}-2\sigma_{X}\sqrt{\left(1-e^{-2R}\right)\left(\sigma_{\hat{X}}^{2}-{\left(\sigma_{X}e^{-(R+R_{c})}-\sqrt{P}\right)}_{+}^{2}\right)} \end{array}$$

*and*

(55) $$\bar{D}\left(R,R_{c},P|W_{2}^{2}\right):=\sigma_{X}^{2}-\sigma_{X}^{2}\xi^{2}\left(R,R_{c}\right)+{\left(\sigma_{X}-\sqrt{P}-\sigma_{X}\xi \left(R,R_{c}\right)\right)}_{+}^{2}.$$

In view of (48), Theorems 4 and 5 imply that

(56) $$D\left(R,R_{c},P|\phi_{KL}\right)\geq \underline{D}\left(R,R_{c},2\sigma_{X}^{2}\left(1-e^{-P}\right)|W_{2}^{2}\right)$$

and

(57) $$D\left(R,R_{c},P|W_{2}^{2}\right)\leq \bar{D}\left(R,R_{c},\nu \left(P\right)|\phi_{KL}\right),$$

where

(58) $$\nu \left(P\right):=\log\frac{2\sigma_{X}^{2}}{{\left(2\sigma_{X}^{2}-P\right)}_{+}}.$$

It is thus of considerable interest to see how these induced bounds compare to their counterparts in Theorems 4 and 5, namely

(59) $$D\left(R,R_{c},P|\phi_{KL}\right)\geq \underline{D}\left(R,R_{c},P|\phi_{KL}\right)$$

and

(60) $$D\left(R,R_{c},P|W_{2}^{2}\right)\leq \bar{D}\left(R,R_{c},P|W_{2}^{2}\right).$$

The following result indicates that (56) and (57) are, in general, looser. In this sense, (59) and (60) are nonredundant.

**Theorem** **6.** *For $p_{X}=N\left(\mu_{X},\sigma_{X}^{2}\right)$,*

(61) $$\underline{D}\left(R,R_{c},P|\phi_{KL}\right)\geq \underline{D}\left(R,R_{c},2\sigma_{X}^{2}\left(1-e^{-P}\right)|W_{2}^{2}\right)$$

*and*

(62) $$\bar{D}\left(R,R_{c},P|W_{2}^{2}\right)\leq \bar{D}\left(R,R_{c},\nu \left(P\right)|\phi_{KL}\right).$$

**Proof of Theorem** **6.** In view of the definitions of $\underline{D}\left(R,R_{c},P|\phi_{KL}\right)$ and $\underline{D}\left(R,R_{c},2\sigma_{X}^{2}\left(1-e^{-P}\right)|W_{2}^{2}\right)$, for the purpose of proving (61), it suffices to show

(63) $$\left[\sigma \left(P\right),\sigma_{X}\right]\subseteq \left[{\left(\sigma_{X}-\sqrt{2\sigma_{X}^{2}\left(1-e^{-P}\right)}\right)}_{+},\sigma_{X}\right]$$

and

(64) $$\sigma_{\hat{X}}^{2}-{\left(\sigma_{X}e^{-(R+R_{c})}-\sqrt{2\sigma_{X}^{2}\left(1-e^{-P}\right)}\right)}_{+}^{2}\geq \sigma_{\hat{X}}^{2}-\sigma_{\hat{X}}^{2}e^{-2(R+R_{c}+P-\psi \left(\sigma_{\hat{X}}\right))}$$

for $\sigma_{\hat{X}}\in \left[\sigma \left(P\right),\sigma_{X}\right]$. Invoking (4) with $p_{\hat{X}}=N\left(\mu_{X},\sigma \left(P\right)\right)$ (see also Lemma 1 for the expressions of the Kullback–Leibler divergence and the squared Wasserstein-2 distance between two Gaussian distributions) yields

(65) $${\left(\sigma_{X}-\sigma \left(P\right)\right)}^{2}\leq 2\sigma_{X}^{2}\left(1-e^{-P}\right),$$

from which (63) follows immediately. Note that (64) is trivially true when $e^{-(R+R_{c})}\leq \sqrt{2(1-e^{-P})}$. When $e^{-(R+R_{c})}>\sqrt{2(1-e^{-P})}$, it can be written equivalently as

(66) $$\sqrt{2(1-e^{-P})}\geq e^{-(R+R_{c})}\left(1-e^{-(P+\frac{\sigma_{X}^{2}-\sigma_{\hat{X}}^{2}}{2\sigma_{X}^{2}})}\right).$$

Since $e^{-(R+R_{c})}\leq 1$ and

(67) $$1-e^{-(P+\frac{\sigma_{X}^{2}-\sigma_{\hat{X}}^{2}}{2\sigma_{X}^{2}})}\leq 1-e^{-(P+\frac{\sigma_{X}^{2}-\sigma^{2}\left(P\right)}{2\sigma_{X}^{2}})}$$

for $\sigma_{\hat{X}}\in \left[\sigma \left(P\right),\sigma_{X}\right]$, it suffices to show

(68) $$\sqrt{2(1-e^{-P})}\geq 1-e^{-(P+\frac{\sigma_{X}^{2}-\sigma^{2}\left(P\right)}{2\sigma_{X}^{2}})}.$$

According to the definition of σ(P),

(69) $$P=\log\frac{\sigma_{X}}{\sigma (P)}+\frac{\sigma^{2}\left(P\right)-\sigma_{X}^{2}}{2\sigma_{X}^{2}}.$$

Substituting (69) into (68) gives

(70) $$\sqrt{2(1-e^{\log\frac{\sigma (P)}{\sigma_{X}}-\frac{\sigma^{2}\left(P\right)}{2\sigma_{X}^{2}}+\frac{1}{2}})}\geq 1-\frac{\sigma (P)}{\sigma_{X}}.$$

We can rewrite (70) as

(71) τ(β)≥0,

where

(72) $$\tau \left(\beta \right):=1-2\beta e^{-\frac{\beta^{2}}{2}+\frac{1}{2}}+2\beta -\beta^{2}$$

with $\beta :=\frac{\sigma (P)}{\sigma_{X}}$. Note that β∈[0,1]. We have

(73) $$\begin{array}{cc} \frac{d\tau (\beta )}{d\beta } & =-2e^{-\frac{\beta^{2}}{2}+\frac{1}{2}}+2\beta^{2}e^{-\frac{\beta^{2}}{2}+\frac{1}{2}}+2-2\beta \\ \; & \leq -2(1-\beta^{2})+2-2\beta \\ \; & =-2(1-\beta )\beta \\ \; & \leq 0. \end{array}$$

Since τ(1)=0, it follows that τ(β)≥0 for β∈[0,1], which verifies (71) and consequently proves (64).

Now, we proceed to prove (62), which is equivalent to

(74) $$\bar{D}\left(R,R_{c},2\sigma_{X}^{2}\left(1-e^{-P}\right)|W_{2}^{2}\right)\leq \bar{D}\left(R,R_{c},P|\phi_{KL}\right).$$

Since $\bar{D}\left(R,R_{c},P|\phi_{KL}\right)=\bar{D}\left(R,R_{c},{\left(\sigma_{X}-\sigma \left(P\right)\right)}^{2}|W_{2}^{2}\right)$, it suffices to show

(75) $${\left(\sigma_{X}-\sigma \left(P\right)\right)}^{2}\leq 2\sigma_{X}^{2}\left(1-e^{-P}\right),$$

i.e.,

(76) $$P\geq \log\frac{2\sigma_{X}^{2}}{\sigma_{X}^{2}-\sigma^{2}\left(P\right)+2\sigma_{X}\sigma \left(P\right)}.$$

Substituting (69) into (76) and rearranging the inequality yields

(77) $$\log\frac{\sigma_{X}^{2}-\sigma^{2}\left(P\right)+2\sigma_{X}\sigma \left(P\right)}{2\sigma_{X}\sigma \left(P\right)}\geq \frac{\sigma_{X}^{2}-\sigma^{2}\left(P\right)}{2\sigma_{X}^{2}},$$

which is indeed true since

(78) $$\begin{array}{cc} \log\frac{\sigma_{X}^{2}-\sigma^{2}\left(P\right)+2\sigma_{X}\sigma \left(P\right)}{2\sigma_{X}\sigma \left(P\right)} & \overset{\left(a\right)}{\geq }1-\frac{2\sigma_{X}\sigma \left(P\right)}{\sigma_{X}^{2}-\sigma^{2}\left(P\right)+2\sigma_{X}\sigma \left(P\right)}\\ \; & =\frac{\sigma_{X}^{2}-\sigma^{2}\left(P\right)}{\sigma_{X}^{2}-\sigma^{2}\left(P\right)+2\sigma_{X}\sigma \left(P\right)}\\ \; & \geq \frac{\sigma_{X}^{2}-\sigma^{2}\left(P\right)}{2\sigma_{X}^{2}}, \end{array}$$

where (*a*) is due to $\log z\geq 1-\frac{1}{z}$ for z>0. This completes the proof of (62). □

**Figure 1:**
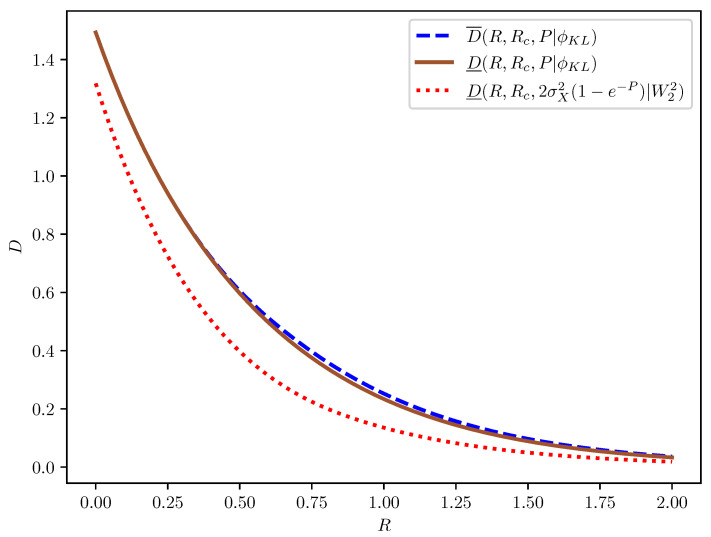
Illustrations of $\bar{D}\left(R,R_{c},P|\phi_{KL}\right)$, $\underline{D}\left(R,R_{c},P|\phi_{KL}\right)$, and $\underline{D}\left(R,R_{c},2\sigma_{X}^{2}\left(1-e^{-P}\right)|W_{2}^{2}\right)$ for $p_{X}=N\left(0,1\right)$, $R_{c}=0$, and P=0.1.

**Figure 2:**
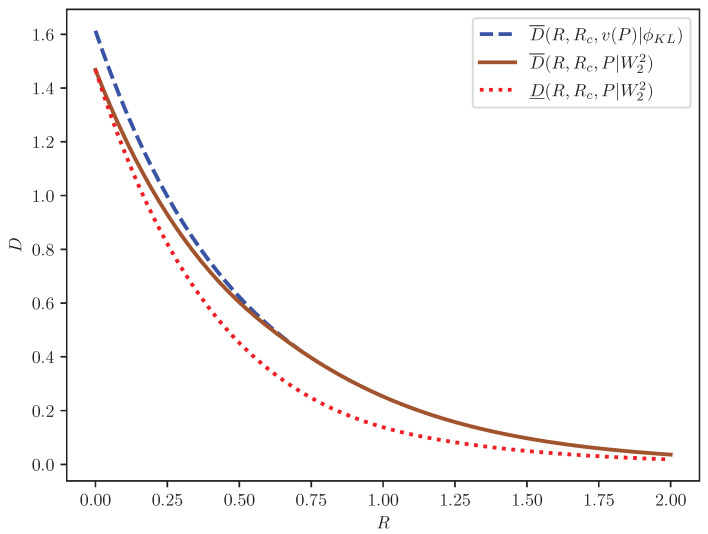
Illustrations of $\bar{D}\left(R,R_{c},\nu \left(P\right)|\phi_{KL}\right)$, $\bar{D}\left(R,R_{c},P|W_{2}^{2}\right)$, and $\underline{D}\left(R,R_{c},P|W_{2}^{2}\right)$ for $p_{X}=N\left(0,1\right)$, $R_{c}=0$, and P=0.1.

It can be seen from Figure 1 that $\underline{D}\left(R,R_{c},2\sigma_{X}^{2}\left(1-e^{-P}\right)|W_{2}^{2}\right)$ is indeed a looser lower bound on $D(R,R_{c},P|\phi_{KL})$ as compared to $\underline{D}\left(R,R_{c},P|\phi_{KL}\right)$, and the latter almost meets the upper bound $\bar{D}\left(R,R_{c},P|\phi_{KL}\right)$. Similarly, Figure 2 shows that $\bar{D}\left(R,R_{c},\nu \left(P\right)|\phi_{KL}\right)$ is indeed a looser upper bound on $D(R,R_{c},P|W_{2}^{2})$ as compared to $\bar{D}\left(R,R_{c},P|W_{2}^{2}\right)$, especially in the low-rate regime, where the latter has a diminishing gap from the lower bound $\underline{D}\left(R,R_{c},P|W_{2}^{2}\right)$.

The fact that the bounds in Theorems 4 and 5 are nonredundant when examined through the connection in (48) serves as evidence of their non-triviality. Consequently, further improvements will likely require exploring deeper properties of the Kullback–Leibler divergence and squared Wasserstein-2 distance.

## 4. Conclusions

In this work, we have established a constrained variant of Talagrand’s transportation inequality. This result reveals a fundamental link between the information-theoretic performance limits of perception-aware lossy source coding under the Kullback–Leibler divergence-based and squared Wasserstein-2 distance-based perception measures. Moreover, it provides an organizational framework for assessing existing bounds in this setting. We believe that similar approaches could be applied to other perception measures. More broadly, the interplay between transportation inequalities and rate-distortion-perception theory presents a rich avenue for further exploration, with promising implications for both theoretical advancements and practical applications.

## Data Availability

Data is contained within the article.
